# P19 Burden of illness and costs among female patients with uncomplicated urinary tract infection in England

**DOI:** 10.1093/jacamr/dlad077.023

**Published:** 2023-08-02

**Authors:** Mark Wilcox, Dave Heaton, Aruni Mulgirigama, Ashish V Joshi, Viktor Chirikov, Daniel C Gibbons, David Webb, Xiaocong Marston, Myriam Alexander, Fanny S Mitrani-Gold

**Affiliations:** University of Leeds & Leeds Teaching Hospitals, Leeds, UK; OPEN Health, Marlow, UK; GSK, Brentford, London, UK; GSK, Collegeville, PA, USA; OPEN Health, Bethesda, MD, USA; GSK, Brentford, London, UK; GSK, Brentford, London, UK; OPEN Health, Marlow, UK; OPEN Health, Marlow, UK; GSK, Collegeville, PA, USA

## Abstract

**Background and Objectives:**

Uncomplicated urinary tract infection (uUTI) is usually community-acquired. Data regarding the wider burden of illness from uUTI are scarce. We evaluated the burden of uUTI in female patients in England.

**Methods:**

This retrospective comparative cohort study utilized linked anonymized patient data from 1 January 2017 to 29 February 2020 from the Clinical Practice Research Datalink (CPRD) and English Hospital Episode Statistics databases. All eligible patients were female, aged ≥12 years and had ≥12 months’ CPRD data available pre- and post-index (i.e. ≥24 months total). Patients with an index UTI diagnosis who had received ≥1 oral antibiotic within ±5 days of index formed the uUTI cohort; uUTI episodes were defined as 28 days from index diagnosis. Patients who had visited a primary care setting but did not have a UTI (uUTI or complicated UTI [cUTI]) diagnosis during the study period formed the control cohort and were matched to the index date of uUTI patients. Patients who were hospitalized or visited an accident and emergency department 28 days prior to index, had cUTI and/or had received IV antibiotics as initial therapy were excluded. Burden of illness (index/12 month follow-up all-cause and UTI-related healthcare resource use [HCRU] and costs) was evaluated in a 1:1 age- and Charlson Comorbidity Index (CCI) score-matched cohort.

**Results:**

Overall, 120519 female patients with uUTI and 233670 female control patients (without uUTI) were identified and matched 1:1. Before matching, more of the uUTI cohort patients versus controls were <50 years old, had a higher mean CCI score, and were more often menopausal; region- and practice-level differences were modest (Table 1). HCRU and costs were significantly higher among uUTI patients versus matched controls, notably for primary care, specialist, and accident and emergency visits, and hospital admissions (*P*<0.001, Table 2). After adjusting for patient characteristics, Poisson or negative binomial models showed significant HCRU differences in primary and secondary care for uUTI patients versus matched controls (Table 3).

**Conclusions:**

Patients with uUTI in England had significantly higher burden of illness, consuming more resources, during index uUTI episodes and follow-up, versus matched controls.

**Table 1.** Selected patient-, region-, and practice-level baseline descriptive characteristics and significant risk factors in unmatched and matched uUTI cohort and controls cohort

Variable description/Statistic or category	Unmatched cohort			Matched cohort		
	uUTI cohort (*N*=120519)	control cohort (*N*=233670)	*P* value	uUTI cohort (*n*=103544)	control cohort (*n*=103544)	*P* value
Patient-level						
Age at diagnosis, years, mean (SD)	52.05 (21.64)	50.13 (20.61)	<0.001	51.37 (20.99)	51.37 (20.99)	0.998
Age at diagnosis, *n* (%)						
≥50	55789 (46.3)	113940 (48.8)	<0.001	48575 (46.9)	48575 (46.9)	1
<50	64730 (53.7)	119730 (51.2)		54969 (53.1)	54969 (53.1)	
Charlson comorbidity index, mean (SD)	0.20 (0.46)	0.15 (0.40)	<0.001	0.18 (0.44)	0.18 (0.44)	1
Race/ethnicity, *n* (%)						
Asian or Asian British	5334 (4.4)	13908 (6.0)	<0.001	4668 (4.5)	5997 (5.8)	<0.001
Black or Black British	3444 (2.9)	11004 (4.7)		3031 (2.9)	4841 (4.7)	
Chinese or other group	2072 (1.7)	6665 (2.9)		1771 (1.7)	2940 (2.8)	
Ethnic group not recorded	24566 (20.4)	52005 (22.3)		20961 (20.2)	22870 (22.1)	
White	42238 (35.0)	76268 (32.6)		36291 (35.0)	33831 (32.7)	
Number of hospital admissions in prior year, mean (SD)	0.31 (0.79)	0.25 (1.51)	<0.001	0.30 (0.78)	0.26 (1.49)	<0.001
Number of A&E attendances in prior year, mean (SD)	0.51 (1.30)	0.24 (0.77)	<0.001	0.49 (1.23)	0.25 (0.77)	<0.001
Smoking status, *n (%)*						
Non-smoker	72944 (62.2)	142422 (64.8)	<0.001	62442 (62.1)	63596 (64.8)	<0.001
Ex-smoker	27218 (23.2)	43960 (20.0)		23315 (23.2)	19932 (20.3)	
Smoker	17046 (14.5)	33328 (15.2)		14824 (14.7)	14582 (14.9)	
Menopausal status, yes, *n* (%)	34276 (28.4)	44326 (19.0)	<0.001	30010 (29.0)	19817 (19.1)	<0.001
Obesity, yes, *n* (%)	13975 (11.6)	24070 (10.3)	<0.001	12154 (11.7)	11195 (10.8)	<0.001
Mild/moderate renal impairment, yes, *n* (%)	2282 (1.9)	2914 (1.2)	<0.001	1702 (1.6)	1561 (1.5)	0.013
Controlled diabetes, yes (HgA1C <6.5/7%)	2473 (2.1)	4535 (1.9)	0.025	2040 (2.0)	2284 (2.2)	<0.001
Region-level, *n* (%)						
Quintiles of index of multiple deprivation						
Q1 (least deprived)	27749 (23.0)	52222 (22.3)	<0.001	23968 (23.1)	23160 (22.4)	<0.001
Q2	24277 (20.1)	47162 (20.2)		20752 (20.0)	20710 (20.0)	
Q3	22992 (19.1)	45665 (19.5)		19604 (18.9)	20351 (19.7)	
Q4	22765 (18.9)	46673 (20.0)		19527 (18.9)	20667 (20.0)	
Q5 (most deprived)	22658 (18.8)	41762 (17.9)		19635 (19.0)	18574 (17.9)	
Missing	78 (0.1)	186 (0.1)		58 (0.1)	82 (0.1)	
Rural-urban area classification						
Rural	15883 (13.2)	28791 (12.3)	<0.001	13695 (13.2)	12918 (12.5)	<0.001
Urban	104636 (86.8)	204879 (87.7)		89849 (86.8)	90626 (87.5)	
Practice-level, *n* (%)						
Practice size						
Small (<8000 patients)	31762 (26.4)	64233 (27.5)	<0.001	27592 (26.6)	28472 (27.5)	<0.001
Medium (8000–11000 patients)	26189 (21.7)	48852 (20.9)		22684 (21.9)	21788 (21.0)	
Large (>11000 patients	62222 (51.6)	120585 (51.6)		53261 (51.4)	53284 (51.5)	
Missing	346 (0.3)	0 (0.0)		7 (0.0)	0 (0.0)	

Percentage values do not add up to 100% for ethnicity as these data were not available for all patients and mixed ethnicity is not shown due to limited interpretability.

A&E, accident and emergency; HgA1C, glycated haemoglobin; Q, quintile.

**Table 2.** All-cause HCRU and costs in matched female patients with uUTI versus control patients in England

Statistic or category	Matched cohort		
	uUTI patient cohort (*n*=103544)	control patient cohort (*n*=103544)	*P* value
HCRU, mean number of events per patient (SD)			
Index uUTI episode (28 days)			
Number of all-cause hospital admissions	0.05 (0.24)	0.02 (0.20)	<0.001^a^
Number of all-cause admissions to HDU/ITU	0.00 (0.03)	0.00 (0.02)	<0.001^a^
Number of all-cause A&E attendances	0.08 (0.38)	0.02 (0.19)	<0.001^a^
Number of all-cause specialist consultations	0.31 (1.00)	0.17 (0.75)	<0.001^a^
Number of all-cause primary care (GP) consultations	1.49 (1.35)	0.26 (0.73)	<0.001^a^
12 month follow-up			
Number of all-cause hospital admissions	0.51 (1.32)	0.24 (1.59)	<0.001^a^
Number of all-cause admissions to HDU/ITU	0.01 (0.10)	0.00 (0.07)	<0.001^a^
Number of all-cause A&E attendances	0.57 (1.48)	0.26 (0.84)	<0.001^a^
Number of all-cause specialist consultations	3.59 (7.43)	1.98 (5.14)	<0.001^a^
Number of all-cause primary care (GP) consultations	7.29 (7.01)	3.12 (4.84)	<0.001^a^
Mean costs, £ (SD)			
Index uUTI episode (28 days), £ (SD)			
Cost of all-cause hospital admissions	72.88 (630.52)	13.03 (219.61)	<0.001^b^
Cost of all-cause admissions to HDU/ITU	6.36 (635.20)	1.41 (148.44)	<0.001^b^
Cost of all-cause A&E attendances	10.16 (55.45)	2.74 (25.98)	<0.001^b^
Cost of all-cause specialist consultations	21.39 (77.33)	11.98 (59.11)	<0.001^b^
Cost of all-cause primary care (GP) consultations	49.27 (44.64)	8.47 (24.16)	<0.001^b^
12 month follow-up			
Cost of all-cause hospital admissions	606.88 (2156.88)	163.22 (994.44)	<0.001^b^
Cost of all-cause admissions to HDU/ITU	42.96 (1541.75)	23.65 (1420.95)	<0.001^b^
Cost of all-cause A&E attendances	70.66 (202.14)	30.65 (109.36)	<0.001^b^
Cost of all-cause specialist consultations	245.58 (470.27)	140.48 (375.38)	<0.001^b^
Cost of all-cause primary care (GP) consultations	240.71 (231.17)	102.97 (159.77)	<0.001^b^

Univariable negative binomial general linear model—this is due to the over dispersion in the count data.

Mann–Whitney *U*-Test.

A&E, accident and emergency; HDU, high dependency unit; ITU, intensive treatment unit.

**Table 3.** Poisson or negative binomial models of the association between uUTI status and HCRU in primary and secondary care, after adjusting for patient characteristics (that remained unbalanced after matching)

Variable/Level	28 day uUTI episode			12 month follow-up		
	RR	CI	*P* value	RR	CI	*P* value
All-cause hospital admissions						
Patient group						
uUTI patient	*Reference level*					
Control patient	0.38	0.356–0.397	<0.001	0.46	0.451–0.466	<0.001
All-cause admissions to high dependency/intensive treatment unit						
Patient group						
uUTI patient	*Reference level*					
Control patient	Results not available			0.49	0.424–0.558	<0.001
All-cause A&E attendances						
Patient group						
uUTI patient	*Reference level*					
Control patient	0.31	0.296–0.326	<0.001	0.52	0.508–0.524	<0.001
All-cause specialist consultations						
Patient group						
uUTI patient	*Reference level*					
Control patient	0.56	0.552–0.574	<0.001	0.52	0.508–0.526	<0.001
All-cause primary care (GP) consultations						
Patient group						
uUTI patient	*Reference level*					
Control patient	0.17	0.170–0.175	<0.001	0.40	0.400–0.407	<0.001

A&E, accident and emergency; RR, risk ratio.

